# Economic Evaluation of a Geriatric Oncology Clinic

**DOI:** 10.3390/cancers14030789

**Published:** 2022-02-03

**Authors:** Shabbir M. H. Alibhai, Zuhair Alam, Ronak Saluja, Uzair Malik, Padraig Warde, Rana Jin, Arielle Berger, Lindy Romanovsky, Kelvin K. W. Chan

**Affiliations:** 1 Department of Medicine, University Health Network, Toronto, ON M5G 2C4, Canada; zuhairalam@gmail.com (Z.A.); ronak.saluja@mail.utoronto.ca (R.S.); uzairmalik@rcsi.ie (U.M.); arielle.berger@uhn.ca (A.B.); lindy.romanovsky@sinaihealth.ca (L.R.); 2 Department of Medicine, University of Toronto, Toronto, ON M5G 2C4, Canada; 3 Institute of Health Policy, Management, and Evaluation, University of Toronto, Toronto, ON M5G 2C4, Canada; 4 Radiation Medicine Program, Princess Margaret Cancer Centre, University Health Network, Toronto, ON M5G 2C4, Canada; padraig.warde@rmp.uhn.ca; 5 Department of Nursing, Princess Margaret Cancer Centre, University Health Network, Toronto, ON M5G 2C4, Canada; rana.jin@uhn.ca; 6 Department of Medical Oncology and Hematology, Sunnybrook Odette Cancer Centre, Toronto, ON M5G 2C4, Canada; kelvin.chan@sunnybrook.ca; 7 Canadian Centre for Applied Research in Cancer Control, Toronto, ON M5G 2C4, Canada

**Keywords:** comprehensive geriatric assessment, economic evaluation, geriatric oncology, treatment decision making, aged, clinic assessment

## Abstract

**Simple Summary:**

There is increasing evidence supporting geriatric assessment (GA) prior to starting cancer treatment in older adults. However, GA is not widely available. One reason may be the lack of persuasive economic data demonstrating its value. We performed an economic evaluation of an academic geriatric oncology clinic and included 152 patients aged 65 years and older who underwent a GA in the pre-treatment setting. We carefully calculated the costs of the proposed treatment (prior to the GA), costs of the GA and associated recommendations, costs of the final treatment, and overall value. We found a GA saved CAD 7387 per patient seen. Extensive sensitivity analyses supported our finding that a GA is economically attractive and should be implemented more widely.

**Abstract:**

Geriatric assessment (GA) is supported by recent trials and guidelines yet rarely implemented due to a lack of resources. We performed an economic evaluation of a geriatric oncology clinic. Pre-GA proposed treatments and post-GA actual treatments were obtained from a detailed chart review of patients seen at a single academic centre. GA-based costs for investigations and referrals were calculated. Unit costs were obtained for surgical, radiation, systemic therapy, laboratory, imaging, physician, nursing, and allied health care (all in 2019 Canadian dollars). A six-month time horizon and government payer perspective were used. Consecutive patients aged 65 years or older (n = 152, mean age 82 y) and referred in the pre-treatment setting between July 2016 and June 2018 were included. Treatment plans were modified for 51% of patients. Costs associated with planned treatment were CAD 3,655,015. Costs associated with GA and related interventions were CAD 95,798. Final treatment costs were CAD 2,436,379. Net savings associated with the clinic were CAD 1,122,837, or CAD 7387 per patient seen. Findings were robust in multiple sensitivity analyses. Combined with mounting trial data demonstrating the clinical benefits of GA, our data can inform a strong business case for geriatric oncology clinics in health care environments similar to ours, but additional studies in diverse health care settings are warranted.

## 1. Introduction

The majority of patients who develop cancer in industrialized nations are older adults (≥65 years). Older adults with cancer face unique challenges due to decreased physiologic reserves, comorbidity, functional impairments, and cognitive impairments. In addition, the oncologic evidence base is usually limited. These factors challenge treatment decision making and lead to the potential for both over- and under-treatment [1].

Geriatric assessment (GA) allows for a more holistic evaluation of the patient and is recommended by several international authorities, including the International Society of Geriatric Oncology (SIOG) [2], the American Society of Clinical Oncology (ASCO) [3], and the National Comprehensive Cancer Network [4]. GA is associated with the detection of important conditions that are missed during the typical oncologic evaluation and results in a median of 28% of treatment decisions being modified, with about two-thirds of modifications being reductions in treatment intensity [5]. Based on emerging randomized trial evidence, GA and management are associated with reduced unplanned health care service use during cancer treatment [6,7,8].

However, the implementation of GA is not widespread, with two of the major barriers being lack of resources (primarily specialist availability) and costs [9,10], leading to limited access to geriatric oncology clinics where GA is typically performed [10].

Since a substantial proportion of treatment modifications after a GA result in less intensive treatment, potential cost savings from avoiding over-treatment may make a compelling business case for the more widespread use of GA. However, there are no published data on potential cost savings associated with GA. If geriatric oncology clinics are associated with cost savings, this can strengthen the business case to establish them and obtain the multiple benefits observed in clinical trials. Therefore, our objective was to perform an economic evaluation of GA in an academic geriatric oncology clinic.

## 2. Materials and Methods

### 2.1. Study Overview and Design

We conducted a retrospective descriptive costing study for patients seen in an academic geriatric oncology clinic. We calculated the costs associated with proposed oncologic treatment plans (prior to GA), the costs of the GA and associated recommendations, and the costs of the final oncologic treatment plans. Extensive sensitivity analyses were performed around all major assumptions to ensure our findings were robust.

Consecutive patients with cancer aged 65 years and older, in the pre-treatment setting, who were referred to the Older Adults with Cancer Clinic (OACC) at the Princess Margaret Cancer Centre in Toronto, Canada, were potentially eligible. Although our clinic opened in July 2015, we restricted this analysis to patients seen between July 2016 and June 2018, representing years 2 and 3 of the clinic. This was for two reasons. First, in year 1, the clinic focused on patients aged 75 years or older with genitourinary cancer and few patients were seen in the pre-treatment setting. Second, we wanted to minimize possible learning effects during the inception phase of the clinic. Net costs were calculated by adding the proposed initial treatment costs minus costs associated with GA and ancillary care (see below for details) and costs of the final treatment plan. The study was approved by the institutional research ethics board, with a waiver for informed consent from patients.

### 2.2. Overview of Geriatric Assessment at Our Centre

At our centre, as described previously, the GA is performed by a clinical nurse specialist and a geriatric medicine specialist [11,12]. It typically requires 1–1.5 h. Eight domains (comorbidity, medications, functional status, fall risk, social support, nutrition, mood, and cognition) are assessed along with vulnerability screening and physical performance testing. Details are summarized in Supplementary Table S1. Recommendations for both oncologic treatment decision making as well as optimization of geriatric domains are made to the referring oncologist. The final treatment plan is decided by the referring oncologist or multidisciplinary tumour board. There is no separate geriatric oncology tumour board.

### 2.3. Treatment Plans and Ancillary Geriatric Care

Initial treatment plans were based on clinical notes in the electronic record, and were abstracted independently by two trained research assistants (ZA, UM). A third reviewer (SMHA) resolved all cases of disagreement. The proposed treatment plan information included modality (e.g., surgery, radiation, and/or systemic therapy), systemic agents, doses, and sequencing (e.g., combined or sequential), as well as treatment intent. Basic sociodemographic and oncologic clinical information was also obtained.

The final treatment plan was determined based on electronic chart review, radiation treatment plans, and discharge summaries. If no active treatment was given over the next six months, treatment was considered to be best supportive care (BSC).

Based on the geriatric oncology team’s recommendations and chart review, we documented each follow-up encounter (telephone or in-clinic) along with additional investigations and referrals. Investigations (laboratory, radiologic, and referrals) were further categorized into those implemented by the OACC team (which were assumed to have occurred in 100 percent of cases) or recommended by the OACC team for other providers to consider and implement, in which case we assumed they were implemented 60% of the time [13,14]. The cost perspective was that of the provincial government, which pays for all treatment-related costs including investigations, medications, surgical procedures, radiation treatment, hospital admissions, and systemic therapies.

### 2.4. Treatment Costs

Detailed costs for two main categories were considered; these were treatment-related and geriatric oncology clinic-related costs. Out of pocket costs were not considered. Costs for each component were gathered in Canadian dollars and were adjusted to 2019 prices using the Consumer Price Index [15]. The time horizon for costing was 6 months from the time of the initial consultation date. Cost data and their sources are summarized in Table 1.

Treatment-related costs included 4 categories:

Surgical costs. Data for average costs for a surgical admission for the specific procedure were obtained from the Ontario Case Costing Initiative (OCCI) [16]. This provided the mean cost for the specific procedure based on per-stay costs (not including physician fees) in all participating hospitals in Ontario. The procedure cost provided by the OCCI includes both direct costs (i.e., nursing, operating room, intensive care unit, inpatient imaging, pharmacy, and laboratory costs) and indirect costs (overhead expenses related to running of the hospital). In cases where the exact surgical procedure was not listed in the OCCI or costing information was not provided to comply with freedom of information directives, the closest matching procedure with reported costs was extracted based on expert input.

Surgeon billings were obtained from published reimbursement schedules for specific procedure codes from the Ontario Ministry of Health and Long-Term Care (MOHLTC). Anesthesiology fees were based on specific procedure codes as well as time-based units that were reimbursed by the MOHLTC [18]. Time estimates for each procedure were obtained from operating room records at our hospital (personal communication, D. Wijeysundera, 5 February 2020). Where too few procedures were performed to allow data to be released, the time for the procedure was estimated by an expert surgical oncologist in the relevant discipline.

Radiation costs. These were estimated from published data by Yong et al. [17]. Those authors used an activity-based costing model and captured all relevant radiation-related costs in 5 categories in Ontario. From these costs for specific procedures, we obtained dose- and fraction-specific radiotherapy costs for curative and palliative radiation and applied these to each radiation treatment planned for, or delivered to, our patients. Where specific radiation doses and schedules were not specified, we consulted internal guidelines at the Princess Margaret Cancer Centre for recommended doses and schedules per disease site and stage.

Systemic therapy. For each systemic therapy, unit costs were obtained from the Sunnybrook Odette Cancer Centre pharmacy. A 28-day cost was then calculated for each patient using their prescribed treatment schedule accounting for body surface area. The final systemic therapy costs included in the analysis were reported as six 28-day cycles of therapy for all patients irrespective of diagnosis or treatment intent. Actual drug doses were captured (i.e., to determine if dose reduction was ordered) and factored into costs. Only one line of systemic therapy was considered, and early discontinuation for progression was not considered.

BSC. The net BSC cost was assumed to be zero, as all patients were assumed to receive BSC in either the initial or final treatment plan.

### 2.5. Costs Associated with GA and Ancillary Geriatric Care

Physician fees for consultation and follow-up care over the next six months were based on actual billings for each study patient based on the physician reimbursement schedule from the MOHLTC. Costs for nursing were based on hourly wages with an estimate of 45 min per new in-clinic assessment, 15 min per telephone follow-up, and 0 min for in-clinic follow-up. Laboratory and radiologic investigations were costed based on specific reimbursement schedules from the MOHLTC. Facility costs for computed tomography and magnetic resonance imaging, which were part of a global hospital budget from the MOHLTC, were obtained from the hospital radiology department (personal communication, Ms. Jane Chen, 1 May 2019). Referrals to other physicians were costed from the physician reimbursement schedule of the MOHLTC. Referrals to allied health professionals (e.g., physiotherapists) or community agencies (e.g., for home personal support workers) were based on hourly wages for publicly funded community providers and estimates of typical provider time for providing similar services (see Supplementary Table S2).

### 2.6. Other Key Assumptions

When multiple initial treatment plans were considered, we consulted local clinical practice guidelines to determine the preferred initial treatment plan and recorded the other option as an alternate.

We assumed changes from initial to final treatment plan were solely due to GA and related recommendations. Both assumptions were examined in sensitivity analyses (see below). Additional assumptions are listed in Supplementary Table S3 along with the potential impact on our results.

### 2.7. Statistical Analyses

Baseline characteristics of the cohort were described using means and standard deviations for continuous data and counts or proportions for categorical data.

### 2.8. Determining Cost Impact of Geriatric Assessment

To determine the net benefit of GA, we calculated the net difference between the initial proposed treatment plan and the final treatment plan and added the costs associated with GA and ancillary geriatric care. An example is provided in the Box.

### 2.9. Sensitivity Analyses

We performed an extensive set of sensitivity analyses to examine the robustness of our findings, including: (1) costs associated with alternate initial treatments; (2) radiation therapy costs were increased or decreased by 25%; (3) proportion of initial treatment plans modified after GA were varied from 0 to 100%; (4) ratio of treatment modifications that were intensified compared with a reduction in intensity or a change to BSC were varied from 0 to 100%; (5) duration of systemic therapy of three or nine months vs. the standard six months; (6) outlier analysis of cost savings; (7) attribution of treatment change to GA; (8) analysis by initial treatment intent. Further details are provided in the Supplementary Materials.

## 3. Results

### 3.1. Study Flow and Patient Characteristics

**Figure 1:**
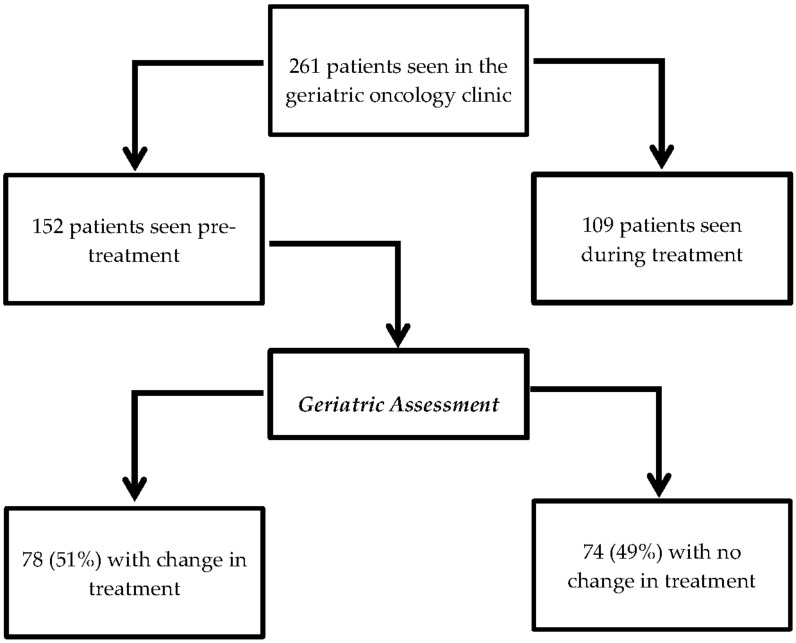
Study flow chart.

A total of 261 patients were seen during the study period, 152 of whom were seen in the pre-treatment setting (Figure 1). One patient was referred twice. Patients had a median age of 82, 60% were male, and 60% had a performance status of 0–1. Treatment intent was curative in 60.9%. Additional clinical and geriatric baseline characteristics are shown in Table 2.

**Figure 2:**
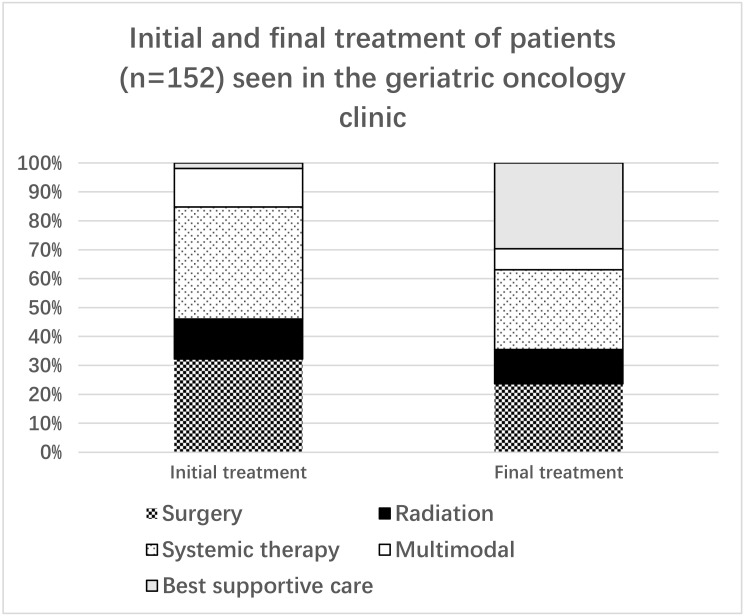
Initial and final treatments for patients included in the study.

Treatment plans were modified in 78 patients (51%) and remained unchanged in 74 (49%). Over 96% of the modified treatment plans resulted in a reduction in treatment intensity (42%) or best supportive care only (54%). The breakdown of initial and final treatment is shown in Figure 2.

### 3.2. Net Costs

The cost of the initial treatment plans for all 152 patients was CAD 3,655,015. The cost of the final treatment plans for all 152 patients was CAD 2,436,379. The cost associated with the OACC appointment and resulting subsequent interventions and follow-up visits was CAD 95,798. The net saving associated with the clinic was therefore CAD 1,122,837 or CAD 7387 per patient, considering all 152 patients irrespective of any change in final treatment. Box 1 provides an illustrative costing example for a sample patient. Supplementary Figure S1 illustrates the distribution of net costs for all 152 patients.

Box 1 **Sample Case**

ID #10977-year-old maleRectal cancer






**Initial treatment plan: Total mesorectal excision, neoadjuvant radiation (no chemotherapy)**






Costs
SurgeryCAD 33,593.00Total mesorectal excision
RadiationCAD 11,008.0050 Gy in 25 fractions

Total
**CAD 44,601.00**








**Final treatment plan: Palliative radiation only**








Costs
Radiation
**CAD 6042.53**
20 Gy in 5 fractions






**OACC costs: CGA + clinic follow up + RN phone call = CAD 477.80**








**Cost analysis**





Cost of initial treatmentCAD 44,601.66


Minus cost of final treatment−CAD 6042.53


Minus OACC costsCAD 477.80



**Cost savings**

**CAD 50,166.39**


Abbreviations: CGA = comprehensive geriatric assessment; OACC = Older Adults with Cancer Clinic; RN = registered nurse

### 3.3. Sensitivity Analyses

Findings were robust for all eight sensitivity analyses. If the cost of initial treatment was altered to reflect alternate proposed treatments, cost savings per patient varied from CAD 6575 to CAD 8199. Varying radiation therapy costs by +/−25% was associated with cost savings per patient of CAD 7094 to CAD 7681. A threshold of 4% or fewer treatment plans being modified was required to reduce the cost savings per patient to 0. The proportion of treatment modifications that led to greater treatment intensity would have to increase from the observed 3% to 54% to reduce the cost savings per patient to 0. Changing the duration of systemic therapy to 3 or 9 months was associated with cost savings per patient of CAD 6483 to CAD 8291, respectively. Eliminating the top 10% of cost savings in an outlier analysis reduced the cost savings per patient to CAD 3542. The proportion of treatment plans modified after a GA that were due to other factors would have to be above 92% for the GA to no longer be cost saving. Finally, cost savings appeared to be higher for patients treated with curative intent (CAD 9091 per patient) compared with those treated with palliative intent (CAD 425 per patient). The impact of additional assumptions on our results is shown in Supplementary Table S3.

## 4. Discussion

We conducted an economic evaluation of a consecutive group of 152 older adults with cancer referred prior to treatment initiation to an academic geriatric oncology clinic. We calculated the costs associated with the proposed treatment plan, added costs associated with the geriatric oncology assessment and related investigations, and subtracted the costs of the final treatment plan to determine the net cost of the geriatric oncology clinic. We found that the clinic was associated with a substantial cost saving of CAD 7387 per patient, primarily due to a reduction in treatment intensity and greater use of best supportive care alone. This cost savings was achieved even though approximately half the patients seen had no change in treatment after the GA.

Prior studies of GA have demonstrated numerous benefits, including the detection of additional conditions such as comorbidity, functional limitations, and cognitive impairment; reduction in treatment toxicity; improved quality of life; and a median of 28% of treatment plans being modified after the GA [3,5,8,17,18]. About two-thirds of the treatment modifications led to reduced treatment intensity. Although both our rate of treatment modifications and the ratio of de-intensified:intensified therapy are at the upper end of estimates from the prior literature, sensitivity analyses demonstrated the robustness of our findings in less optimistic scenarios. What is novel about our findings is that we have demonstrated cost savings associated with a GA in this setting, primarily achieved through a reduction in treatment intensity or a move to BSC alone for a substantial proportion of patients. At our academic cancer referral center, it was rare for treatment intensity to be increased after a GA, reflecting the local culture and approach to care as a tertiary care academic referral center. Although prior studies of GA in the oncology setting have not examined costs directly, our findings are not surprising given the reduction in treatment intensity that has been reported in multiple studies, and one group recently demonstrated reductions in unplanned health care use with GA in a randomized trial [8]. Altogether, these findings provide a strong business case to invest resources into geriatric oncology clinics or into providers who can perform GA in more oncology centers.

In non-cancer settings, GA has also been shown to be cost effective in several settings, including various inpatient, outpatient specialist, and outpatient primary care settings [19,20]. One important reason for our program to be cost saving, in contrast to most other geriatric services, is due to the reduction in treatment intensity of cancer. Cancer treatments are quite expensive and systemic therapy costs in particular are substantial and increasing over time [21]. Other geriatric programs have focused on improving function or quality of life and avoiding falls. These benefits, although important on many levels, may not lead to cost savings unless associated with reduced hospital or long-term care admissions.

### 4.1. Strengths and Limitations

This study has several strengths, including being the first to comprehensively determine the costs and savings associated with GA, the rigorous determination of all relevant costs, including post-GA recommendations for investigations, referrals, and rehabilitation, and the inclusion of a consecutive sample of patients typical of a geriatric oncology practice. Several important limitations must also be considered. First, it was a single academic center study with a modest small sample size in a public health care system. The generalizability to non-academic, non-government payer-based systems is uncertain and confirmatory studies in those settings are required. Second, costs were attributed retrospectively based on chart review, which may not have been complete. Third, a number of important assumptions were made as mentioned previously. Many of these assumptions were examined in extensive sensitivity analyses and found not to affect our findings. Fourth, we did not explicitly consider a reduction in treatment toxicity or unplanned health care use as benefits of GA. Two recently published RCTs of GA and co-management demonstrated 10–20% reductions in severe toxicity with systemic therapy in the metastatic setting along with reductions in unplanned health care use [6,7]. Incorporating these would have made GA even more cost effective in our study. Fifth, patient adherence to therapy, particularly radiation or systemic therapy, was not systematically captured, although we did vary the duration of systemic therapy in a sensitivity analysis. Sixth, downstream treatments, progression, and overall survival were not captured in order to keep the study and assumptions manageable. Whether incorporating these components would increase or decrease net savings is unclear. Seventh, we did not examine either short-term or long-term quality of life; maintaining quality of life is an important consideration for many older adults with cancer. Finally, although treatment plans were modified for over 50% of patients seen in the geriatric oncology clinic, we did not attempt to evaluate if the final treatment was the most appropriate for the patient. Importantly, the final treatment decision was left to the discretion of the referring oncologist and/or tumor board. Although the proportion of treatments that were modified in our clinic was high relative to the median of 28% from a recent meta-analysis [5], our sensitivity analysis demonstrated that only 4% of treatments would need to be modified after the GA to preserve its cost effectiveness. Given the variations in oncologic practice and costs in different health care systems, this emphasizes the need to validate our findings in different clinical settings.

### 4.2. Future Considerations

Given the aforementioned limitations, it is important to perform similar studies in different settings. Exploring other barriers to implementing GA is also important, including examining other models of care. For example, frailty screening, universal referral, or nurse-led geriatric assessments may be more or less costly than the present model [22,23]. Analyzing hospitalizations/re-admissions or treatment complication rates as an additional dimension of possible economic benefit of GA should also be explored.

## 5. Conclusions

A geriatric oncology clinic is cost saving in our setting, primarily because of a substantial reduction in treatment intensity after GA. These data can inform a strong business case in health care environments similar to ours, but additional studies in diverse health care settings are warranted. Our findings add to the growing evidence base in favor of implementing GA more widely in the oncology setting.

## Figures and Tables

**Table 1:** Cost components, sources, and related assumptions.

Component	Source	Assumptions
**Surgical procedures**	OCCI costing tool (average cost per case) [16]	Specific procedure was not always reported in notes
**Systemic therapy**	Sunnybrook Health Sciences Centre oncology pharmacy, with costs adjusted for body surface area	6 cycles, 28 days each, no early discontinuation/switching
**Radiotherapy**	Published Ontario costing data [17]	Extrapolated from costs for breast and prostate (Yong et al.)
**Laboratory tests, imaging**	Government reimbursement schedules [18] Hospital costs for CT and MRI from the department of medical imaging	Standard cost for all blood tests Standard cost for all plain X-rays and ultrasounds Hospital-based costs for computed tomography and magnetic resonance imaging
**Physicians**	Government billing guides for consultations by most specialists [18] Hospital-based estimates of in/out time for time-based anesthesiologist fees (billed at government-specified unit-based rates)	No costs for trainees
**Nurse, social worker, interpreter, physiotherapy, occupational therapy**	Hourly wages	15 min per telephone follow-up No nursing costs in follow up clinic visits
**Miscellaneous (outpatient rehabilitation, exercise program, falls prevention program, Lifeline, Meals on wheels, Wheels Trans, etc.)**	Various (see Supplementary Table S2)	See Supplementary Table S2

**Table 2:** Baseline characteristics of patients seen pre-treatment and included in the cost analysis (n = 151 *).

Characteristic	Distribution ^#^
**Age, median, years (range)**	82 (61–96)
**Gender, male**	91 (60%)
**ECOG performance status**	
0	28 (19)
1	62 (41)
≥2	61 (40)
**Comorbidity** ^^^	
Low	52 (34%)
Moderate	62 (41%)
High	37 (25%)
**VES-13 ≥ 3** (*n* = 149)	132 (89%)
**Disease Site**	
Gastrointestinal	49 (32.5%)
Genitourinary	36 (23.8%)
Head and neck	24 (15.9%)
Leukemia, lymphoma	14 (9.3%)
Gynecological	8 (5.3%)
Thoracic	8 (5.3%)
Breast	4 (2.6%)
Skin (not melanoma)	3 (2.0%)
Melanoma	3 (2.0%)
Myeloma	1 (0.7%)
Other	1 (0.7%)
**Treatment intent**	
Curative	92 (60.9%)
Palliative	59 (39.1%)
**Geriatric domains**	
Dependent in 1 or more IADLs	63 (42%)
Abnormal Physical Performance ^§^	104 (68.9%)
Medication Optimization Issues	109 (72%)
Increased Falls Risk	99 (66%)
Social Supports (Vulnerable or Poor)	39 (26.8%)
Nutrition	
At risk	57 (37.7%)
Malnourished	8 (5.3%)
Mood	
Depressed	23 (15%)
Unable to assess fully	8 (5%)
Cognition	
Abnormal	46 (30%)
Borderline/requires further testing	20 (13%)

NOTE: ECOG = Eastern Cooperative Oncology Group; IADL = Instrumental Activities of Daily Living; VES = Vulnerable Elders Survey 13-item (a score of 3 or higher indicates increased vulnerability/frailty). * One patient was referred twice but baseline characteristics were only included once. ^#^ Although the general cut-off was age 65 and older, an exception was made for one frail and medically complex patient aged 61. ^^^ Based on Charlson comorbidity index score and clinical judgement. ^§^ Based on grip strength and short physical performance battery.

## Data Availability

Restrictions apply to the availability of these data. Data were obtained from the University Health Network and are available from the authors with the permission of the Research Ethics Board of the University Health Network.

## Supplementary Materials

The following are available online at https://www.mdpi.com/article/10.3390/cancers14030789/s1, Supplementary methods: sensitivity analyses, Table S1: components of geriatric assessment, Table S2: list of costs, Table S3: list of assumptions, Figure S1: histogram of net costs.
