# Supplementary material for: Economic Evaluation of a Geriatric Oncology Clinic
Source: Cancers (Basel). 2022 Feb 3;14(3):789. doi: 10.3390/cancers14030789 (PMC8833958; doi:10.3390/cancers14030789)
Supplement: Supplementary file 1 [file cancers-14-00789-s001.zip › cancers-1573302-supplementary.pdf]

Economic evaluation of a geriatric oncology clinic

Supplemental materials

1. Supplemental methods: sensitivity analyses
2. Supplemental Table S1 – components of geriatric assessment
3. Supplemental Table S2 – list of costs
4. Supplemental Table S3 - list of assumptions
5. Supplemental Figure S1 - histogram of net costs

Supplemental methods

Sensitivity Analyses

We performed a series of sensitivity analyses to examine the robustness of our findings. We examined: 1) Alternate proposed regimen; 2) Radiation therapy costs were increased or decreased by 25%; 3) Proportion of initial treatment plans modified after geriatric assessment (GA); 4) Proportion of treatment plans where treatment was reduced in intensity or changed to supportive care; 5) Duration of systemic therapy 3 vs 6 vs 9 months; 6) Cost outlier analysis; Attribution of treatment change to GA; 8) Analysis by treatment intent.

1) Alternate proposed regimen. For a number of patients, more than one initial treatment regimen was proposed or considered. It was not always possible to determine which was the intended treatment option from the patient's medical record and obtaining clarification from the treating clinician after the GA would be subject to recall bias. In such instances, we consulted local clinical practice guidelines to determine the intended treatment option. To examine the potential effects of this approach, we identified all patients in which more than one initial regimen was suggested by the treating oncologist. We varied the cost of initial treatment upwards by 25% and downwards by 25% to reflect variability in treatment regimen costs and determined the effect on our findings.

2) Radiation therapy costs were increased or decreased by 25%. Net radiation costs were varied upwards by 25% and downwards by 25% for both the initial treatment and final treatment to determine the effect on our findings.

3) Proportion of initial treatment plans modified after GA. In our study we identified a relatively large 57% of treatment plans that were modified. In comparison, in the literature the median rate is 28%, with a range of 8-54% [1]. We calculated the threshold of patients whose treatment plans would need to be modified, assuming a similar type of modification in the final treatment plan as we observed in our full dataset (i.e. no change to the proportion in whom the final treatment was more intensive or less intensive than the initial treatment plan, and no change in the proportion whose treatments were modified to best supportive care).

4) Proportion of treatment plans where treatment was reduced in intensity or changed to supportive care. In our study we identified that the vast majority of patients (94%) whose treatment plans were modified had a final treatment plan that was less intensive than the original plan. This compares with about 65% in the literature[1]. We varied this in a sensitivity analysis by calculating the average net cost for a patient whose treatment was intensified (n=4 in our original dataset) and the average net cost for a patient whose treatment intensity was reduced (n=74; we included both reduction in treatment intensity and use of best supportive care only in this category). While keeping the proportion of treatment plans that were changed constant, we varied the ratio of intensification to reduction in treatment intensity to determine if there was a threshold at which GA was no longer cost saving.

5) Duration of systemic therapy. In our base case analysis, we assumed each patient would be treated for 6 months. In this sensitivity analysis, we varied the duration of initial systemic

therapy treatment (including cytotoxic chemotherapy, targeted therapy, and immunotherapy) to 3 months and 9 months to determine the effect on our findings.

6) Cost outlier analysis. Examining the distribution of net costs per patient in our clinic, the distribution suggested some extreme values in terms of cost savings from treatment plan changes (e.g. from intensive multimodality therapy to best supportive care). To examine the potential influence of these outliers on our overall findings, we eliminated the most extreme decile of cost savings and recalculated our cost savings per patient.

7) Attribution of treatment change to GA. We had assumed that all treatment plan changes that occurred after the GA were due to the assessment. In a sensitivity analysis we varied the proportion of cases in whom treatment plans were modified after GA to be attributed to the GA from 0 to 100%.

8) Analysis by treatment intent. Since decision making and modifications to treatment plans after GA may vary by treatment intent, we divided the sample into those whose initial treatment intent was curative vs palliative and reran our analyses.

References

1. Hamaker ME, Te Molder M, Thielen N, et al: The effect of a geriatric evaluation on treatment decisions and outcome for older cancer patients - A systematic review. J Geriatr Oncol 9:430-440, 2018

Supplemental Table S1: Components of Geriatric Assessment

**Geriatric Domain**	**Instrument**
Vulnerability	Vulnerable Elders Survey 13-item
Comorbidity	Charlson Comorbidity Index (CCI); modified by clinical judgment
Medication optimization	Detailed medication review by clinician
Social support	Structured questions about living situation, paid and unpaid supports
Function	i. Older Adults Resources and Services Instrumental Activities of Daily Living instrument (if abnormal then Katz Activities of Basic Living done)<br>ii. Grip strength for upper extremities<br>iii. Short physical performance battery for lower extremities
Falls risk	Single question on whether ≥1 falls in the last six months
Nutrition	i. Weight loss in the past 6 months<br>ii. Measurement of body mass index
Cognition	Mini-Cog
Mood	Patient Health Questionnaire 2 or 9 item*

* Patient Health Questionnaire (PHQ) 2 and 9; part-way through the study time period routine care in the clinic was modified so that the PHQ-2 was done first and the PHQ-9 was done only if the PHQ-2 was positive.

Supplemental Table S2: List of costs by category

Category of costs

BLOOD TESTS	Item	Source	Code	Cost
	Complete blood count	MOHLTC laboratory medicine schedule Nov 17, 2017	L393	$3.98
	Creatinine	same	L067	$1.28
	Electrolytes	same	L053, L061, L204, L226	$5.06
	Ferritin	same	L329	$2.97
	Glucose, Fasting	same	L111	$1.28
	TSH	same	L341	$3.58
	Urine culture	same	L634	$10.34
	Vitamin B12	same	L345	$3.58

CHEMOTHERAPY	Drug/Regimen	Sample Dosage/schedule	Phamacy dose	Pharmacy cost
	5-Fluorouracil	1000 mg/m^2/day, IV, days 1-4 and 29-32	50mg/ml	$160.90/100ml
	Abiraterone	1000 mg PO qd	500mg	$59.18
	Azacitidine	75 mg/m^2 SC qd days 1-7	100mg/vial	$599.99/vial
	Bevacizumab	5 mg/kg IV on day 1	25mg/ml	$2171.57/16ml
	Bicalutamide	50 mg PO qd	50mg tabs	$48.30/30 tabs
	Capecitabine	1000mg/m^2 PO BID days 1-14	500mg	$1.53
	Carboplatin	Carboplatin AUC 5 IV day 1	10mg/ml	$44.85/ 60ml
	Cetuximab	250 mg IV weekly for 24 weeks	100mg	$364.62
	Chlorambucil	6 mg/m^2 PO days 1 to 14	2mg tabs	$38.72/25 tabs
	Cisplatin	100mg/m^2 IV on day 1, q21d	1mg/ml	$681.53/100ml
	Cyclophosphamide	Cyclophosphamide 100 mg f5d	1000mg/vial	$92.39/50ml
	Degarelix	Initial Dose; 240 mg SC day 1 of month 1	120mg/vial	$727.95
	Denosumab	120 mg SC q4weeks	120mg/1.7ml inj	$637.37/vial
	Dexamethasone	0.5 mg PO qd	10mg/ml	$12.00/10ml
	Docetaxel	75 mg/m^2, IV on day 1, q21d f total 6 cycles	10mg/ml	$2047.13/16ml
	Doxorubicin	60 mg/m^2 q3weeks	2mg/ml	$50.00/100ml
	Enzalutamide	160 mg PO qd (continuous)	40mg cap	$3696.13/120 caps
	Exemestane	25 mg PO qd	25mg tabs	$39.79/30 tabs
	Everolimus	10 mg PO qd (continuous)	10mg tabs	$6414.00/30 tabs
	FOLFIRI	Irinotecan 180 mg/m^2	20mg/ml	$61.82/25ml
		Leucovorin 400 mg/m^2	10mg/ml	$689.43/50ml
		Fluorouracil 400 mg/m^2 IV day 1	50mg/ml	$160.90/100ml
	FOLFIRINOX	Oxaliplatin 85mg/m^2 IV day 1	5mg/ml	$146.06/40ml
		Leucovorin 400mg/m^2	10mg/ml	$689.43/50ml
		Irinotecan 180mg/m^2	20mg/ml	$61.82/25ml

	Fluorouracil 400mg/m^2	50mg/ml	$160.90/100ml
Gefitinib	250mg PO QD	250mg tabs	$2319.95/30 tabs
Gemcitabine	1000mg/m^2 IV days 1, 8 and 15 q28d	38mg/ml	$46.36/2000mg
Goserelin	10.8 mg q90d	10.8mg	$1,271
Ibrutinib	420 mg po qd	140mg caps	$9,067.73/90
Irinotecan	180 mg/m^2	20mg/ml	$61.82/25ml
Letrozole	2.5 mg PO qd	2.5mg tabs	$217.62/30
Leucovorin	400 mg/m^2	10mg/ml	689.43 /50ml
Leuprolide	22.5 mg q3m	22.5mg inj	$1,129.91
Mitomycin	10 mg/m^2, IV, on days 1 and 29	20mg/ vial	$414.92/ vial
Nivolumab	3 mg/kg, IV, on day 1 q14d	100mg/10ml	$2151.12/vial
Obinutuzumab	1000 mg IV day 1, 8, 15	25mg/ml	$5728.06/40ml
Paclitaxel	80 mg/m^2 Iv d1 q7d every 12 weeks	6mg/ml	$34.47/50ml
Trastuzumab	4 mg/kg	440mg/vial	$2958.01/vial
Pembrolizumab	2 mg/kg IV day 1 q21d	25mg/ml	$4400.00/4ml
Prednisone	10 mg PO qd (cont)	5mg tabs	$17.30/1000
R-CHOP	Prednisone 100 mg PO qd days 1-5,	50mg tabs	$9.00/100
	Rituximab 375 mg/m^2 IV day 1	120mg/ml SC inj	$3090.04/15ml
	Vincristine 1.4 mg/m^2 IV day 1	1mg/ml	$153.00/5ml
	Doxorubicin 50 mg/m^2 IV day 1	2mg/ml	$50.00/100ml
	Cyclophosphamide 750 mg/m^2 IV day 1	1000mg/vial	$92.39/50ml
Mini-R-CHOP	Rituximab 375 mg/m^2 IV	120mg/ml SC inj	$3090.04/15ml
	Rituximab 1400 mg SC on day 1	120mg/ml SC inj	$3090.04/15ml
	Prednisone 50 mg PO qd days 1-5	50mg tabs	$9.00/100
	Vincristine 0.7 mg/m^2 IV day 1	1mg/ml	$153.00/5ml
	Doxorubicin 25 mg/m^2 IV day 1	2mg/ml	$50.00/100ml
	Cyclophosphamide 375 mg/m^2 IV day 1	1000mg/vial	$92.39/50ml
Siltuximab	11 mg/kg IV for one dose on day 1 q21d		$364.62
Sofarenib	400 mg PO BID continuous	200mg tabs	$5704.92/120 tabs
Sunitinib	50 mg PO qd, days 1-28 q6weeks	50mg caps	$7611.31/28
Tamoxifen	20 mg daily	10mg	$10.50/30

IMAGING	**Item**	**Source**	**Code**	**Cost**
	Arterial leg dopplers	MOHLTC SOB, G7	J193	$36.35
	Bone mineral density test	MOHLTC SOB, D15	X146	$103.20
	CT Head without contrast	UHN case costing database	n/a	Proprietary
	CT Head with contrast	UHN case costing database	n/a	Proprietary
	CT Chest	UHN case costing database	n/a	Proprietary
	MRI Head	UHN case costing database	n/a	Proprietary
	X-ray Chest (PA and lateral)	MOHLTC SOB, D9	X090	$21.30
	X-ray Foot	MOHLTC SOB, D6	X069	$21.30
	X-ray Hand	MOHLTC SOB, D6	X054	$21.30

	X-ray Shoulder	MOHLTC SOB, D6	X048	$25.90
MISCELLANEOUS	**Item**	**Source**	**Code**	**Cost**
	Allergy testing	MOHLTC SOB, A41	K399	$29.05
	Electrocardiogram	MOHLTC SOB, J12		$11.0500
	Echocardiogram	MOHLTC SOB, J18	G570/571	$208.8000
	Holter monitor, 48hr	MOHLTC SOB, J14	G682,683,658	$188.6500
	Lifeline	Website (www.lifeline.ca)	$30/month (basic plan)	$180.00
	Meals on Wheels	Website (www.mealsonwheels.ca)	Per meal	$7.00
	Pulmonary Function Test	MOHLTC SOB, H3	J3XX	$119.70
	Sleep study	MOHLTC SOB, J88	J896	$468.2500
	Wheel Trans	Website (http://www.ttc.ca/Fares_and_passes/index.jsp)	Patient costs are same as public transit one-way adult fare. System costs are different.	$3.25
PERSONNEL	**Individual**	**Source**	**Code/Comment**	**Cost**
	Anaesthesia	MOHLTC SOB, GP58	Procedure and unit-based	Variable
	Cardiology	MOHLTC SOB, A47	A605 (consult)	$157.00
	Dietician	Hospital	$45/hour	$45.00
	Endocrinology	MOHLTC SOB, A57	A155 (consult)	$157.00
	ENT	MOHLTC SOB, A99	A245 (consult)	$77.90
	Falls Prevention Program	Hospital	$50/session, 12 sessions	$600.00
	Geriatrician	MOHLTC SOB, A71	A075 (consult)	$175.00
			A775 (CGA, 75 min)	$300.70
			A770 (CGA, 90 min)	$395.00
			A071 (follow up visit)	$70.90
	Geriatric oncology nurse	Hospital	$45/hour; assume 15 min/phone call	$11.25
	Grief Counsellor	Assumed	$50/hour; assume 2 hours	$100.00
	In-Patient Rehabilitation	Rehab program	Average of FY16-17 and FY17-18	$812.50
	Interpreter	Hospital	Hourly	$60.00
	Memory Clinic	Multiple	Consultation by geriatrician, neurologist, psychiatrist plus $50 for allied health assessment	$600.75
	Neurology	MOHLTC SOB, A86	A185 (consult)	$176.35
	Nurse	Hospital	Hourly	$34.50
	Occupational therapist	Hospital	$50/hour; assume 2 hours	$100.00
	Ophthalmology	MOHLTC SOB, A92	A235 (consult)	$82.30
	Palliative Care	MOHLTC SOB, J82	G512 (consult)	$62.75
	Personal Support Worker	Estimate	Hourly	$25.00
	Physiotherapist	Hospital	$50/hour x 4 sessions at home	$200.00
			$50/hour x 10 sessions in clinic	$500.00
	Podiatrist	Estimate		$50.00
	Psychiatry	MOHLTC SOB, A111	A195 (consult)	$199.40
	Respirology	MOHLTC SOB, A120	A475 (consult)	$157.00

	Rheumatology	MOHLTC SOB, A122	A485 (consult)	$157.00
	Social Work	Hospital	$50/hour, assumes 2 hours	$100.00
	Urogynecology	MOHLTC SOB, A91	A205 (consult)	$101.70
	Urology	MOHLTC SOB, A126	A355 (consult)	$80.00
	Vestibular Rehab	Estimate	$60 initial assessment, then $30 per treatment x 12 sessions	$420.00

RADIATION CT simulation, dosimetry/planning, physics/quality assurance, supporting infrastructure, and treatment delivery costs (including nursing and radiation oncologist supervision) extrapolated from Yong et al. (Curr Oncol 2016; 23(3):e228) and Earle et al. (Crit Rev Oncol Hematol 1999; 32:87).

SURGERY Specific procedures are all from individual entries in the Ontario Case Costing Initiative project
https://data.ontario.ca/en/dataset/ontario-case-costing-initiative-occi

Abbreviations
MOHLTC = Ontario Ministry of Health and Long-Term Care
SOB = Schedule of Benefits https://www.health.gov.on.ca/en/pro/programs/ohip/sob/
CGA = comprehensive geriatric assessment

Supplemental Table S3: List of Additional Assumptions And Potential Impact on Analyses/Results

Assumption	*Impact on analyses/results*
Treatment-related	
1. Radiotherapy costs per fraction including cost of radiation technologist, computed tomography simulation, dosimetry/planning, radiation physicist, and nurse review derived from published Ontario-specific prostate and breast data and extrapolated to other sites.	No impact on results as it affected all radiation treatments similarly. Tested in sensitivity analysis #2
2. Whenever details of the exact radiation treatment plan (doses and fractionation schedule) were not specified, we followed local clinical practice guidelines.	No impact on results as it affected all radiation treatments similarly. Radiation costs were varied in sensitivity analysis #2.
3. Procedure costs are only the costs pulled from the Ontario Case Costing Initiative tool. Average physician billings for these procedures were based on government fee schedules for surgeons and anesthetists. The latter included time-based and procedure-based elements. Time per procedure was based on an internal hospital database. When a time estimate was not present for a specific procedure in the database, clinical experts who perform that procedure (e.g. uro-oncologist) were contacted to provide an estimate.	Standard approach to costing. No impact on results.
4. Systemic therapy costs were reported per cycle and we made the blanket assumption of six months of therapy for all patients irrespective of diagnosis or treatment intent. We also assumed the full course of therapy was delivered without interruption, delays, further dose reductions, or early discontinuation.	Simplifying assumption. If this was formally incorporated the direction of effect is uncertain since both groups (treatment unchanged and treatment changed after geriatric assessment) would be affected. Tested in sensitivity analysis #5 with 3 months or 9 months of systemic therapy.
5. We did not attempt to determine if patients expired within the 6-month window of treatment (see prior assumption) if treatment was initiated.	Simplifying assumption. If accounted for, would likely lead to greater cost savings from geriatric assessment as patients whose treatments were modified may have a slightly shorter life expectancy due to less aggressive treatment overall.

6. For all patients who died shortly after being seen in the geriatric oncology clinic and who died prior to starting treatment (n=2), we assumed no costs for either the planned oncologic treatment or final treatment but calculated costs associated with the geriatric oncology clinic.	This was felt to be more conservative than simply excluding these patients, which might make the clinic look more economically attractive than otherwise, and it avoids assumptions around whether the final treatment would have been modified or not. Increases costs of geriatric assessment slightly (i.e. making the geriatric oncology clinic less cost saving).
7. Only one line of treatment was modelled. So if the patient went on to second-line chemotherapy within 6 months this was not captured but the duration of the first-line of treatment was not modified (see assumption #4).	Simplifying assumption. If accounted for, would likely lead to greater cost savings from geriatric assessment as these patients would be less likely to undergo second-line treatment.
8. If more than one treatment plan was being considered but it was unclear what the recommended initial treatment was, we followed local clinical practice guidelines.	Simplifying assumption. Tested in sensitivity analysis #1.
9. We assumed that if the final treatment plan was modified, it was due to the recommendation from the geriatric oncology clinic.	Simplifying assumption. Tested as part of sensitivity analysis #7.
10. Costs of supportive care medications (e.g. antiemetics) were not explicitly considered.	Simplifying assumption. If accounted for, would likely lead to greater cost savings from geriatric assessment as fewer patients underwent active cancer treatment.
Clinic-related	
11. Learners are costed at $0 beyond the time factored into supervising physician billings (which may have included learner time assessing patients) and additional testing ordered.	Simplifying assumption. If accounted for, would fractionally increase costs associated with geriatric assessment but these costs would be counterbalanced by potential reductions in time-based clinician billings. Net effect would be negligible.
12. Follow-up visits are billed as complex medical reassessments by the geriatrician (A071 code). No nurse time is assumed in an in-person follow-up clinic visit.	Simplifying assumption based on discussion with clinic staff, informal observations, and prior nursing staff audit. If accounted for, would fractionally increase costs associated

	with geriatric assessments. Net effect would be negligible.
13. Telephone follow-ups are assumed to require 15 minutes nursing time, no physician time. No cost was factored in for failed telephone contact attempts (e.g. voicemail left). No cost was factored in for discussions between nurse and geriatric oncology clinic physician.	Simplifying assumption based on discussion with clinic staff, informal observations, and prior nursing staff audit. If accounted for, would fractionally increase costs associated with geriatric assessments. Net effect would be negligible.
14. Investigations/Imaging/Referrals were based on the costs for specific items in the costing table. If these were "suggested to be ordered" by the referring oncology team as opposed to actually ordered by the geriatric oncology team, they were costed at 60% of the listed cost, assuming that an implementation rate of 60% of geriatric recommendations is optimistic. In a randomized trial of geriatric assessment, non-geriatric physicians implemented 59% of recommendations [1]. In a large recent randomized trial of geriatric assessment and management in geriatric oncology, 0-91% of recommendations were implemented by oncologists [2]. In a large prospective multicenter study of geriatric assessment, the 3 most common recommendations were implemented 47.6-59.5% of cases [3].	Simplifying assumption based on published geriatric literature and local quality improvement targets. If accounted for, direction of cost effect uncertain as implementation may be lower or higher than 60%. Net effect would be negligible regardless given small cost of geriatric assessment compared to treatment costs (~0.8%).
15. All Investigations/Imaging/Referrals from follow up visits were summed up into one column and not specified by which visit they were ordered/suggested to order in. Additionally, re-referrals (e.g. to physiotherapy) were costed only once under the assumption that the patient did not see them on the first referral.	Simplifying assumption. No impact on results as aggregate costs would be largely unchanged.
16. A single cost was assigned if any blood tests were ordered and was based on the government schedule of benefits for laboratory procedures. The standard blood work panel is assumed to be a complete blood count, electrolytes, creatinine, glucose, and 1/10th cost of a thyroid stimulating hormone (TSH; one of TSH, B12, or iron studies, each of which is similar in cost, was done in about 1 in 10 patients seen in our clinic).	Simplifying assumption. Net effect would be negligible regardless given small cost of investigations as part of geriatric assessment, which itself had a small cost compared to treatment costs.

17. Costs for plain radiography included technical and physician interpretation-based costs. These were from the government schedule of benefits for radiographic procedures. Costs for computed tomography and magnetic resonance imaging had technical costs that were obtained from the imaging department at the hospital whereas physician interpretation fees were from the government schedule of benefits.	Standard costing approach. No effect on results.
18. Clinic-related costs were only captured for 6 months (i.e. if a patient had further follow-up visits after 6 months, these were not captured). This was because our usual practice was to see patients in consultation and once in follow-up during systemic therapy or after local treatment. Fewer than 5% of patients had follow-up visits more than 6 months after the initial visit.	Simplifying assumption. If accounted for, net effect would be negligible given relatively few follow up assessments beyond 6 months and small overall cost of geriatric assessment (and lesser costs of follow-up care) compared to treatment costs.

References

1. Reuben DB, Frank JC, Hirsch SH, McGuigan KA, Maly RC. A randomized clinical trial of outpatient comprehensive geriatric assessment coupled with an intervention to increase adherence to recommendations. *J Am Geriatr Soc*. Mar 1999;47(3):269-76. doi:10.1111/j.1532-5415.1999.tb02988.x
2. Mohile SG, Mohamed MR, Xu H, et al. Evaluation of geriatric assessment and management on the toxic effects of cancer treatment (GAP70+): a cluster-randomised study. *Lancet*. Nov 3 2021;doi:10.1016/S0140-6736(21)01789-X
3. Kenis C, Decoster L, Flamaing J, et al. Adherence to geriatric assessment-based recommendations in older patients with cancer: a multicenter prospective cohort study in Belgium. *Ann Oncol*. Sep 1 2018;29(9):1987-1994. doi:10.1093/annonc/mdy210

Supplemental Figure S1: Histogram of net costs per patient seen

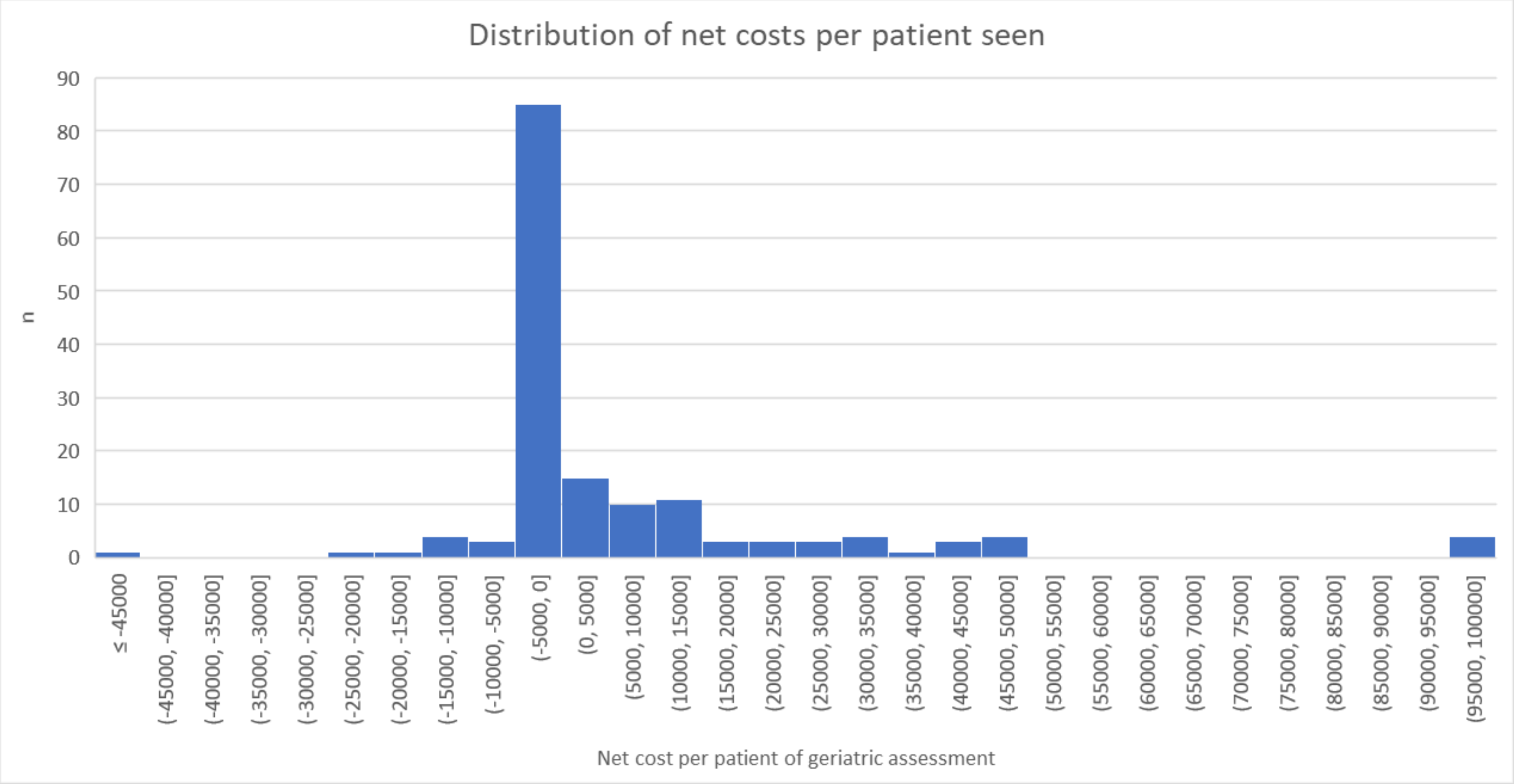

The above costs are net costs for each patient seen, and are calculated based on costs of initial treatment – (costs of final treatment + costs of geriatric oncology clinic visit and related investigations/referrals). For the vast majority of patients, net costs were in the $0 to $5,000 range (i.e. it was slightly more expensive to be seen in the geriatric oncology clinic).
